# Salicylic Acid and Sodium Salicylate Alleviate Cadmium Toxicity to Different Extents in Maize (*Zea mays* L.)

**DOI:** 10.1371/journal.pone.0160157

**Published:** 2016-08-04

**Authors:** Orsolya Kinga Gondor, Magda Pál, Éva Darkó, Tibor Janda, Gabriella Szalai

**Affiliations:** Department of Plant Physiology, Agricultural Institute, Centre for Agricultural Research, Hungarian Academy of Sciences, Martonvásár, Hungary; National Research Council of Italy, ITALY

## Abstract

The role of salicylic acid in Cd tolerance has attracted more attention recently but no information is available on the efficiency of different forms of salicylic acid. The aim was thus to investigate whether both the acid and salt forms of salicylic acid provide protection against Cd stress and to compare their mode of action. Young maize plants were grown under controlled environmental conditions. One group of 10-day-old seedlings were treated with 0.5 mM SA or NaSA for 1 day then half of the pants were treated with 0.5 mM Cd for 1 day. Another group of seedlings was treated with 0.5 mM CdSO_4_ for 1 day without pre-treatment with SA or NaSA, while a third group was treated simultaneously with Cd and either SA or NaSA. Both salicylic acid forms reduced the Cd accumulation in the roots. Treatment with the acidic form meliorated the Cd accumulation in the leaves, while Na-salicylate increased the phytochelatin level in the roots and the amount of salicylic acid in the leaves. Furthermore, increased antioxidant enzyme activity was mainly induced by the acid form, while glutathione-related redox changes were influenced mostly by the salt form. The acidic and salt forms of salicylic acid affected the two antioxidant systems in different ways, and the influence of these two forms on the distribution and detoxification of Cd also differed. The present results also draw attention to the fact that generalisations about the stress protective mechanisms induced by salicylic acid are misleading since different forms of SA may exert different effects on the plants via separate mechanisms.

## Introduction

In plants, cadmium is an easily absorbed and rapidly translocated heavy metal, and causes strong toxicity even at relatively low concentrations. Although maize (*Zea mays* L.) is a relatively Cd-tolerant plant, many toxic symptoms may result if the Cd concentration exceeds a critical level, 7–70 mg/kg or more depending on the soil conditions [1–3]. Cd toxicity is often associated with oxidative stress, caused by the excessive formation of reactive oxygen species (ROS) [4]. The antioxidant system plays an important role in protection against various stressors. The most important non-enzymatic antioxidants are glutathione (reduced form GSH, oxidised form GSSG), ascorbic acid (AsA) and phenolic metabolites. GSH is also the substrate for the synthesis of phytochelatins (PCs), which play a special role in the detoxification of toxic heavy metals [5–6].

Salicylic acid (SA) plays a key role in the signal transduction pathways of various stress responses [7–9]. In one of the first papers demonstrating the protective effect of SA against abiotic stress factors, SA treatment was reported to induce tolerance to copper toxicity in cucumber and tobacco [10]. The ameliorating effect of SA treatment on seed germination and seedling growth was also shown during Pb^2+^ or Hg^2+^ stress in rice [11]. Later, much more attention was attracted by the role of SA in Cd tolerance [12–13].

Although a relatively large number of studies have described the role of SA in stress adaptation processes (mainly using exogenous SA), the results are often conflicting. The effect of SA on various stresses is somewhat ambiguous. An elevated endogenous level of SA was found to intensify the phytotoxicity caused by lead or Cd in *Arabidopsis* plants [14]. SA may cause oxidative stress in plants, partly through the accumulation of hydrogen peroxide [15]. SA was also shown to stimulate the generation of ROS in photosynthetic tissues of *Arabidopsis thaliana* during salt or osmotic stress, thus participating in the development of stress symptoms [16].

The application of SA may either be harmful or provide protection during abiotic stress, depending on the plant species, the concentration used and the mode of application [8]. The kind of SA used is often not specified. Only a few of the papers that mention “SA” treatment indicate whether the acidic form of SA or sodium salicylate (NaSA) was applied. Papers related to SA and stress responses usually give a very general discussion of the mode of action of SA as a protective compound. No data are available on the comparative effects of different SA forms under the same conditions. The inconsistencies among experimental data in the literature may thus be due to the use of different forms of SA and different application modes. The aim was thus to investigate whether the acid and salt forms of SA provide the same protection during Cd stress and whether their mode of action differs. The results clearly demonstrated that different forms of SA may induce very diverse defence mechanisms.

## Materials and Methods

### Plant Material

Sterilized seeds of maize (*Zea mays* L., hybrid Norma) were germinated for 3 days at 26°C, after which six seedlings per beaker were grown in 400 ml modified Hoagland solution [17] at 22/20°C with 16/8-h light/dark periodicity in a Conviron PGR-15 plant growth chamber (Controlled Environments Ltd, Winnipeg, Canada) in the phytotron of the Agricultural Institute, Centre for Agricultural Research, Hungarian Academy of Sciences. The photosynthetic photon flux density was 240 μmol m^-2^ s^-1^, provided by metal halide lamps, with a relative humidity of 75%. After 10 days various treatments were applied. A schematic presentation of the various treatments can be seen in S1 Fig. One group of seedlings was treated with 0.5 mM SA (SigmaUltra; S5922, Sigma Aldrich) or NaSA (Fluka; 71945, Sigma Aldrich) for 1 day (designated as SA pre, NaSA pre, respectively), after which half of the plants were moved on the original growth solution and the second half were treated with 0.5 mM CdSO_4_ for 1 day (SA pre Cd, NaSA pre Cd).

Another group of seedlings was treated with 0.5 mM CdSO_4_ for 1 day without pre-treatment with SA or NaSA, while a third group was treated simultaneously with Cd and either SA or NaSA (SA+Cd, NaSA+Cd). The initial pH of the plant growth solution (5.5) was not changed significantly by NaSA, while SA reduced it to 3.8. The third leaves and roots were collected for analysis as indicated in S1 Fig.

### Measurement of Chlorophyll Content

Chlorophyll content was estimated using a CL-01 Chlorophyll Content Meter (Hansatech Instruments Ltd, King's Lynn, Norfolk, UK) in the youngest fully developed (third) leaves. The readings are given as relative chlorophyll contents using dual wavelength optical absorbance (620 and 940 nm) measurements.

### Chlorophyll-a Fluorescence Induction Measurements

Chlorophyll-a fluorescence quenching analysis was carried out using a pulse amplitude modulated fluorometer (Imaging-PAM M-Series fluorometer; Walz, Effeltrich, Germany). The Fv/Fm parameter was determined on plants previously dark-adapted for 20 minutes, using a 0.8 s saturating pulse (PPFD = 3000 μmol m^-2^ s^-1^) provided by a LED-Array Illumination Unit IMAG-MAX/L (λ = 450 nm). Photosynthesis was then activated using 230 μmol m^-2^ s^-1^ actinic light intensity for 15 min and quenching analysis was performed using 0.8 s saturating pulses applied every 30 s. The quenching parameters were determined under steady state conditions according to the nomenclature described by Klughammer and Schreiber [18].

### Measurement of Cd Content

The Cd contents of the roots and leaves were measured according to Hegedűs et al. [19].

### Measurement of PC and PCS Activity

The PC content and PCS activity were measured as described by Szalai et al. [3] using 750 mg plant material. PCS activity was calculated as the PC produced during 60 min incubation compared with the initial level. The activities were expressed as nkatal g^-1^ protein.

### Measurement of Thiols

200 mg plant material was ground in liquid nitrogen using a mortar and pestle, after which 1 ml of 0.1 M HCl was added. The content of reduced and oxidised thiol forms (Cys, γEC, GSH) was determined according to Gulyás et al. [20]. The activities of γECS and GSHS were measured as described by Brunner et al. [21]. The half-cell reduction potential of the glutathione redox couples (E_GSSG/2GSH_) was calculated using the Nernst equation [22].

### Measurement of MDA

The lipid peroxidation analysis was based on the malondialdehyde (MDA) level [23], which was measured spectrophotometrically at 532 nm, with the subtraction of non-specific absorption at 600 nm. MDA was then quantified using an extinction coefficient of 155 mM^−1^cm^−1^, and expressed as nM g^−1^ fresh weight.

### Measurement of AsA

The plant samples were ground to a fine powder using liquid nitrogen and the AsA was extracted from 200 mg samples with 1.5 ml 1.5% metaphosphoric acid. The AsA content was measured as described earlier by Szalai et al. [24].

### Antioxidant Enzyme Assays

For the analysis of antioxidant enzyme activity, 0.5 g plant material were homogenised in 2.5 mL of ice-cold Tris buffer (0.5 M, pH 7.5) containing 3 mM MgCl_2_ and 1 mM EDTA.

The catalase (CAT; EC 1.11.1.6) activity of the extract was measured spectrophotometrically by monitoring the decrease in absorbance at 240 nm. The reaction mixture contained 0.44 M Tris buffer (pH 7.4), 0.0375% H_2_O_2_ and enzyme extract [17].

The ascorbate peroxidase (APX; EC 1.11.1.11) activity was determined in the presence of 0.2 M Tris buffer (pH 7.8) and 5.625 mM AsA. The reaction was started with 0.042% H_2_O_2_ and the decrease in absorbance at 290 nm was monitored [17].

The guaiacol peroxidase (POD; EC 1.11.1.7) activity was measured at 470 nm as described by Ádám et al. [25]. The reaction mixture consisted of 88 mM Na-acetate buffer (pH 5.5), 0.88 mM guaiacol, 0.0375% H_2_O_2_ and enzyme extract.

The glutathione reductase (GR; EC 1.6.4.2) activity was determined at 412 nm according to Smith et al. [26]. The reaction mixture contained 75 mM Na-phosphate buffer (pH 7.5), 0.15 mM diethylenetriamine-pentaacetic acid, 0.75 mM 5,5’-dithiobis (2-nitrobenzoic acid), 0.1 mM NADPH, 0.5 mM oxidised glutathione and 50 ml plant extract in a total volume of 1 ml.

The glutathione-S-transferase (GST; EC 2.5.1.18) activity was measured by monitoring changes in the absorbance at 340 nm in a mixture containing 72.7 mM Na-phosphate buffer (pH 6.5), 3.6 mM reduced glutathione, 1 mM 1-chloro-2,4-dinitrobenzene and enzyme extract [27].

The activities were expressed in nkatal g^-1^ protein.

### Protein Content

The total protein content was determined according to the method of Bradford [28] using bovine serum albumin as standard.

### Statistical Analysis

The experiments were repeated 3 times and representative data are shown. The results were the means of 5 measurements. The data were statistically evaluated using the standard deviation and *t*-test methods.

## Results

### Cadmium Content

**Fig 1:**
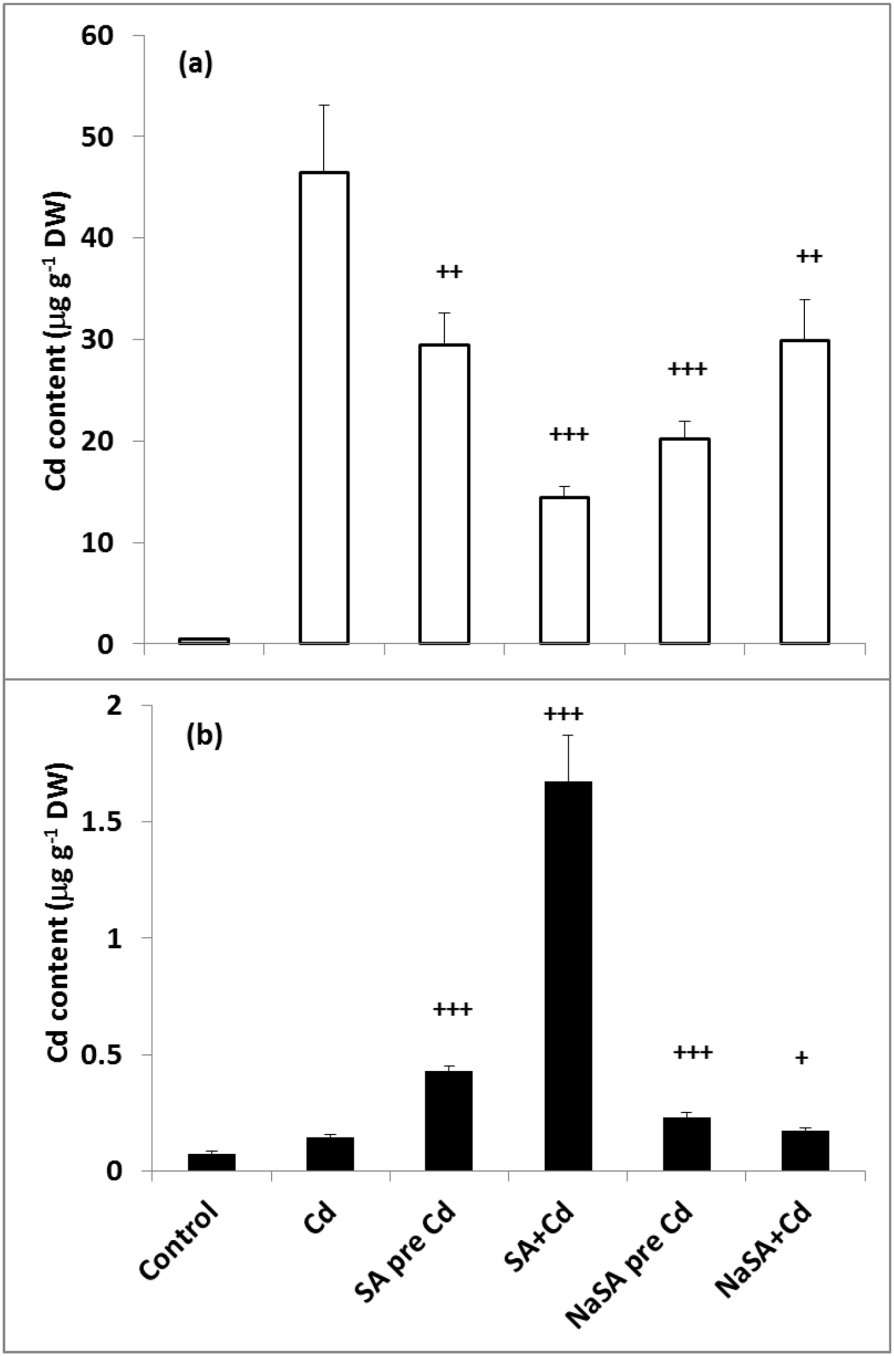
Changes in the Cd content in the roots (a) and leaves (b) of young maize plants after 0.5 mM SA or NaSA treatment during Cd stress. (For details see legend of Table 1).

After one day of exposure to Cd the highest Cd content was measured in the roots of plants given no SA or NaSA treatment (Fig 1a). In plants treated with SA or NaSA the roots contained less Cd than in plants treated with Cd alone; the lowest level of Cd was detected in the roots when SA and Cd were added together (SA+Cd). By contrast, the highest Cd content in the leaves was found in SA+Cd plants (Fig 1b). The leaves of the other SA/NaSA-treated plants accumulated far less Cd, but the lowest Cd level was found in plants given no SA or NaSA. The amount of Cd in the leaves was two orders of magnitude lower than that in the roots.

### Chlorophyll Content and Chlorophyll-A Fluorescence Parameters

Pre-treatment of maize plants with 0.5 mM SA significantly reduced the maximum quantum efficiency of Photosystem 2 (PS2) represented by the Fv/Fm ratio compared with the control (Table 1). This decrease was even more pronounced in the actual quantum yield (Φ_PS2_) and the chlorophyll content. These changes were accompanied by a substantial increase in non-photochemical quenching Y(NPQ). The increase in non-regulated non-photochemical quenching Y(NO) was not significant. When NaSA was used, the decrease in Fv/Fm was significant but not substantial compared with the control plants; furthermore, a slight increase in Φ_PS2_ and a decrease in NPQ could be detected. However, this treatment also reduced the chlorophyll content, which suggests that this is probably not the parameter responsible for the SA-induced changes in the photosynthetic electron transport processes.

**Table 1:** Changes in the chlorophyll-*a* fluorescence parameters and chlorophyll content in the leaves of young maize plants after 0.5 mM SA or NaSA treatment during Cd stress.

	Fv/Fm	Quantum Yield of PS2 (Φ_PS2_)	Y(NPQ)	Y(NO)	Chlorophyll content
Control	0.777±0.006	0.371±0.024	0.386±0.022	0.242±0.013	12.4±1.5
SA pre	0.640±0.049	0.122±0.039	0.644±0.048	0.270±0.039	7.6±1.4
	***	***	***	**	*
NaSA pre	0.764±0.005	0.415±0.009	0.324±0.019	0.255±0.013	8.9±1.6
	***	***	***	*	
Cadmium	0.719±0.013	0.105±0.010	0.656±0.041	0.235±0.025	8.7±0.9
	***	***	***		***
SA pre Cd	0.632±0.045	0.111±0.011	0.577±0.041	0.314±0.071	6.2±1.5
	+++		+++	+++	+++
SA + Cd	0.676±0.042	0.141±0.044	0.527±0.046	0.318±0.062	7.7±1.1
	+++	++	+++	+++	+++
NaSA pre Cd	0.737±0.006	0.421±0.024	0.315±0.022	0.258±0.017	8.1±1.2
	+++	+++	+++	+	
NaSA + Cd	0.737±0.019	0.320±0.051	0.468±0.070	0.246±0.016	8.1±1.2
	+	+++	+++		

*, **, *** significant difference between the control and SA pre-, NaSA pre- or Cd-treated plants at the p < 0.05, 0.01 and 0.001 levels, respectively.

^**+**^, ^**++**^, ^**+++**^ significant difference between plants treated with Cd alone or in combination with SA or NaSA at the p < 0.05, 0.01 and 0.001 levels, respectively.

(SA pre: 0.5 mM SA pre-treatment for 1 day; NaSA pre: 0.5 mM NaSA pre-treatment for 1 day; Cd: 0.5 mM Cd for 1 day; SA pre Cd: 0.5 mM SA pre-treatment for 1 day followed by 0.5 mM Cd stress for 1 day; SA+Cd: addition of 0.5 mM SA and 0.5 mM Cd together for 1 day; NaSA pre Cd: 0.5 mM NaSA pre-treatment for 1 day followed by 0.5 mM Cd stress for 1 day; NaSA+Cd: addition of 0.5 mM NaSA and 0.5 mM Cd together for 1 day)

Treatment of maize plants with Cd also decreased the chlorophyll content, Fv/Fm, and especially Φ_PS2_ (Table 1), while increasing Y(NPQ) but not Y(NO). Plants which were pre-treated with SA showed similar changes; however, pre-treatment with NaSA significantly alleviated the Cd-induced changes in the fluorescence induction parameters. In fact, Φ_PS2_ was slightly, but significantly higher compared with the Cd-treated plants. When SA or NaSA was added at the same time as Cd, the protective effect of NaSA was less pronounced than in plants pre-treated with this compound before exposure to Cd; however, plants treated with SA gave similar results regardless of whether it was added before or simultaneously with Cd (Table 1).

### Phytochelatin Content and Phytochelatin Synthase Activity

**Fig 2:**
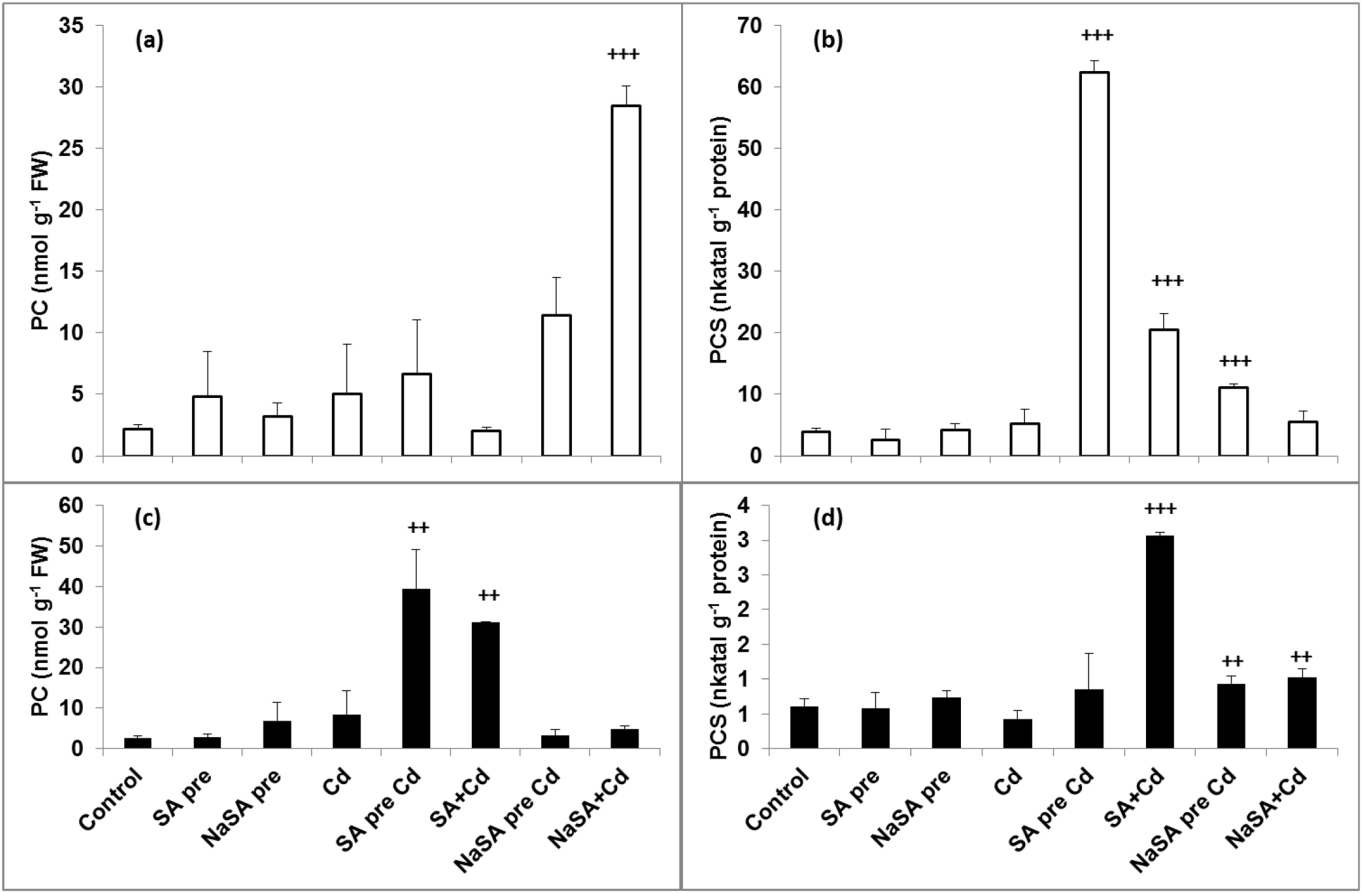
Changes in the PC content and PCS activity in the leaves and roots of young maize plants after 0.5 mM SA or NaSA treatment during Cd stress. (a): PC content in the roots; (b): PCS activity in the roots, (c): PC content in the leaves; (d): PCS activity in the leaves. (For details see legend of Table 1).

Neither SA nor NaSA pre-treatment increased the PC content or enhanced the activity of PCS in the leaves and roots (Fig 2). A rise in the PC level was observed after Cd treatment in the roots of plants pre-treated with NaSA, while this was much more pronounced when NaSA and Cd were added at the same time (Fig 2a). Although the PC content increased substantially in the roots of NaSA-treated plants after Cd addition, the PCS activity only increased slightly and no changes were detected when NaSA and Cd were added together (Fig 2b). In contrast, a substantial increase in PCS activity was detected in the roots of plants pre-treated with SA after Cd addition (Fig 2d), and this was associated with higher PC content in the leaves (Fig 2c). A considerable rise in both PC level and PCS activity was also observed in the leaves when SA and Cd were added together (Fig 2c and 2d).

### Influence of Various SA Treatments on Glutathione Synthesis during Cd Stress

**Fig 3:**
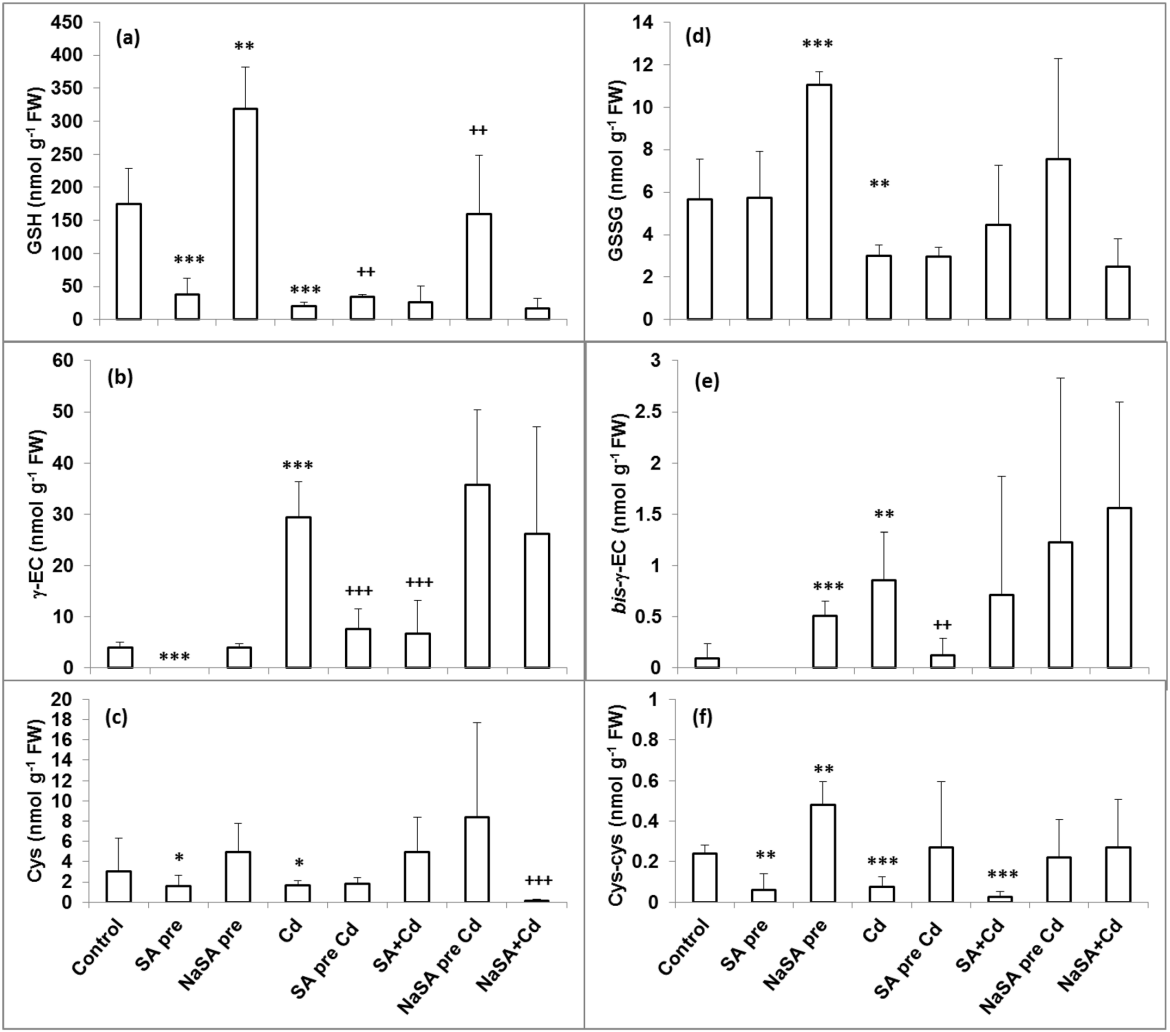
Changes in the GSH (a), γEC (b), Cys (c), GSSG (d), *bis*- γEC (e) and Cys-Cys (f) content in the roots of young maize plants after 0.5 mM SA or NaSA treatment during Cd stress. (For details see legend of Table 1).

**Fig 4:**
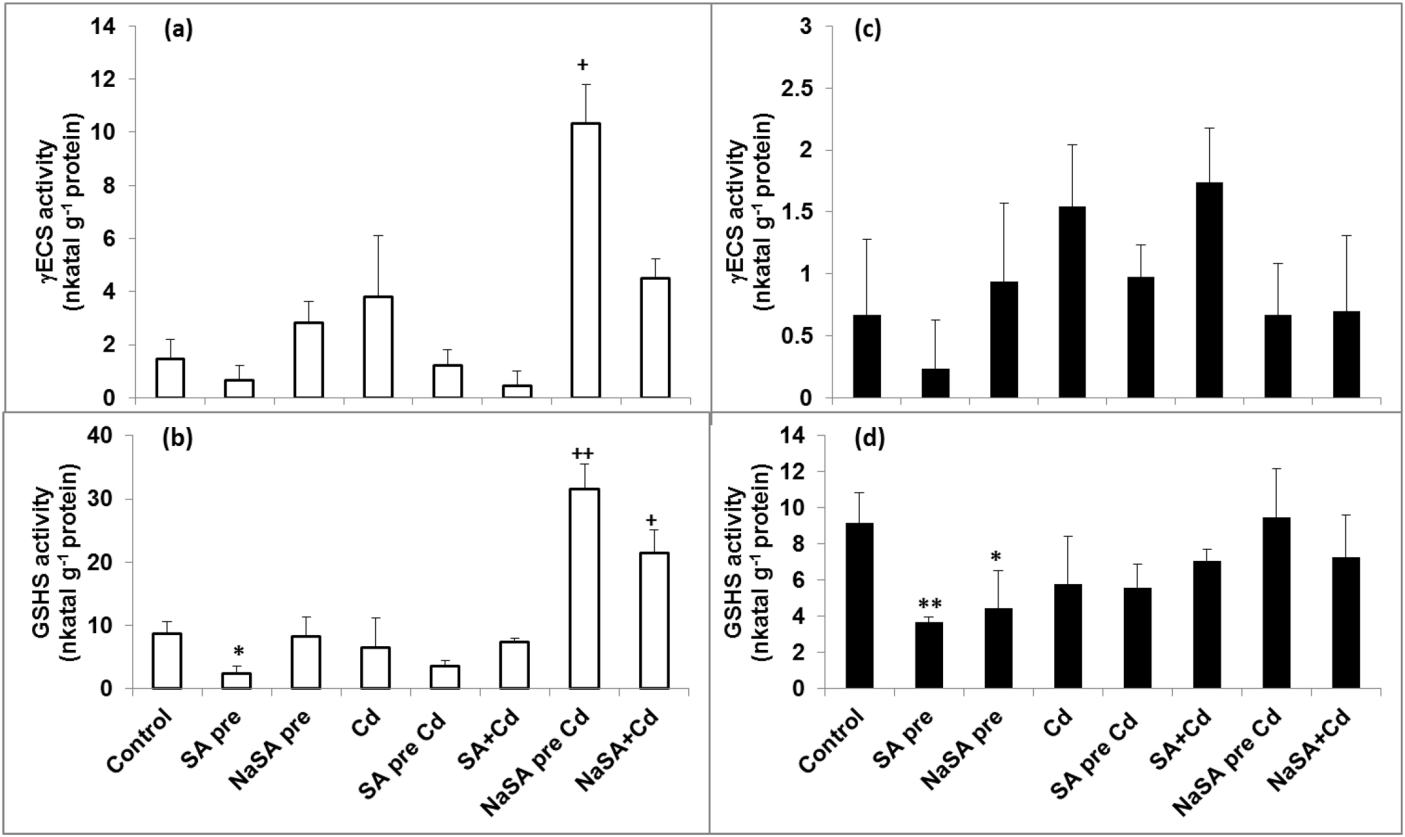
Changes in the γECS and GSHS activity in the leaves and roots of young maize plants after 0.5 mM SA or NaSA treatment during Cd stress. (a): γECS activity in the roots; (b): GSHS activity in the roots; (c): γECS activity in the leaves; (d): GSHS activity in the leaves. (For details see legend of Table 1).

The GSH content increased after NaSA pre-treatment in the roots compared with the control (Fig 3a), while it decreased when the plants were treated with SA. A decrease in GSH could be observed after one day of Cd stress, but in plants pre-treated with NaSA it remained at the control level. The γEC content only increased after Cd addition (Fig 3b), but not in SA-treated plants. No great changes could be observed in the Cys level in the roots (Fig 3c). Both the γECS and GSHS activities only increased when NaSA pre-treatment was followed by Cd addition (Fig 4a and 4b) or when NaSA and Cd were added together.

**Fig 5:**
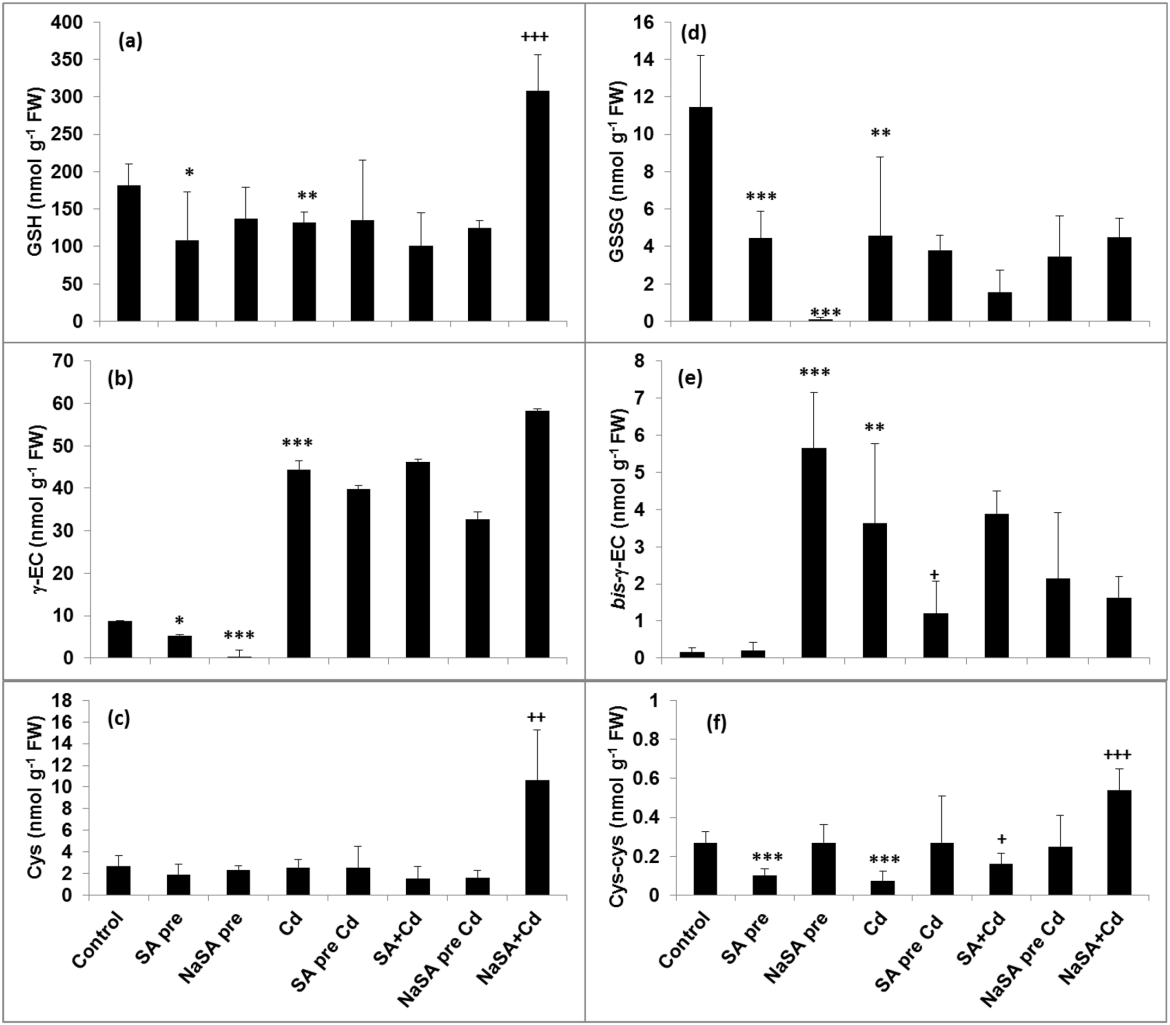
Changes in the GSH (a), γEC (b), Cys (c), GSSG (d), *bis*- γEC (e) and Cys-Cys (f) content in the leaves of young maize plants after 0.5 mM SA or NaSA treatment during Cd stress. (For details see legend of Table 1).

The GSH and Cys levels only increased in the leaves compared with the control (Fig 5a and 5c) when NaSA and Cd were added at the same time, while the amount of γEC rose in all cases after Cd treatment (Fig 5b). Few changes were observed in the enzyme activities in the leaves (Fig 4c and 4d).

### Oxidative Stress

**Fig 6:**
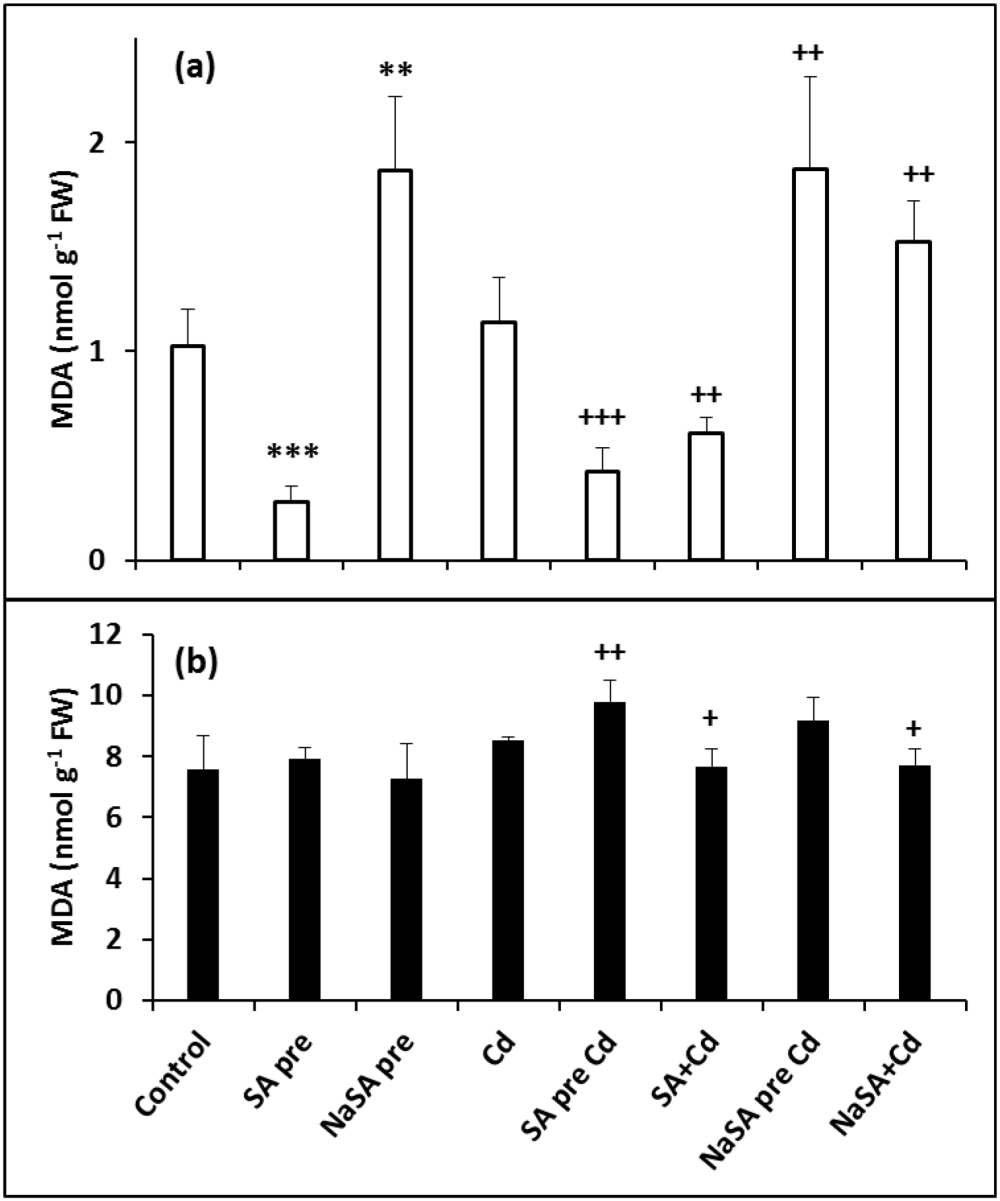
Changes in the MDA content in the roots (a) and leaves (b) of young maize plants after 0.5 mM SA or NaSA treatment during Cd stress. (For details see legend of Table 1).

The extent of oxidative stress was estimated by determining the MDA content, which decreased after SA pre-treatment (Fig 6a) but increased in the roots of plants pre-treated with NaSA compared with the control. Similar tendencies were detected after Cd treatment, with a lower MDA level in SA-treated plants and a higher one after treatment with NaSA compared with the Cd-treated plants. Cd itself did not enhance the oxidative stress in the roots compared with the control, while in the leaves Cd addition only caused a slight elevation of the MDA content in plants pre-treated with SA or NaSA (Fig 6b). Catalases and peroxidases take part in the detoxification of ROS. The catalase (CAT) activity in the roots was ten times higher after SA pre-treatment (Table 2) and six times higher when SA and Cd were added together compared with the control. CAT activity was only doubled by NaSA pre-treatment, but it was four times higher after Cd addition in comparison with control plants. In contrast, no considerable changes could be detected in the leaves. SA pre-treatment raised the guaiacol peroxidase (POD) activity in the roots compared with the control (Table 2), which was much higher when SA and Cd were added together. The POD activity diminished after Cd addition in NaSA pre-treated roots compared with the controls. A slight decrease was also observed when NaSA and Cd were added at the same time. The POD activity in the leaves was not influenced by pre-treatment (Table 2), but it increased after Cd treatment in both non- and pre-treated plants. No changes were found when SA or NaSA were added at the same time as Cd compared with the control leaves.

**Table 2:** Changes in the antioxidant enzyme activities in the leaves and roots of young maize plants after 0.5 mM SA or NaSA treatment and during Cd stress.

	Control	SA pre	NaSA pre	Cd	SA pre Cd	SA + Cd	NaSA pre Cd	NaSA + Cd
**Leaves**								
CAT	71.6 ±21.5	64.9 ± 5.4	41.8 ± 7.3	67.2 ± 26.6	49.7 ± 9.68	44.8 ± 17.7	89.0 ± 40.4	78.6 ± 20.2
			*					
POD	11.1 ± 3.4	13.9 ± 0.9	9.03 ± 1.42	17.7 ± 3.9	20.7 ± 3.1	17.1 ± 5.6	16.3 ± 2.7	15.9 ± 5.8
				*				
GR	0.49 ± 0.18	1.19 ± 0.13	0.58 ± 0.24	0.83 ± 0.15	1.35 ± 0.38	0.78 ± 0.42	0.73 ± 0.11	0.54 ± 0.11
		***		**	+		+	++
GST	1.02 ± 0.16	0.95 ± 0.26	0.85 ± 0.14	0.69 ± 0.31	0.98 ± 0.15	0.64 ± 0.15	0.53 ± 0.23	0.74 ± 0.11
				*				
APX	2.13 ± 0.70	2.71 ± 0.37	1.85 ± 1.19	3.32 ± 0.69	4.33 ± 1.19	5.00 ± 1.51	2.61 ± 0.65	4.62 ± 0.53
				**		+		++
**Roots**								
CAT	161 ± 24	1624 ± 211	360 ± 29	210 ± 17	157 ± 36	1022 ± 444	710 ± 88	289 ± 40
		***	***	*		+		
POD	1671 ± 415	2813 ± 646	1314 ± 346	1622 ± 420	1677 ± 145	4864 ± 450	880 ± 86	1114 ± 233
		*				+++	++	+
GR	7.19 ± 2.64	44.3 ± 18.2	54.1 ± 6.19	22.4 ± 10.7	7.25 ± 2.67	52.3 ± 22.4	14.5 ± 5.7	13.0 ± 4.5
		**	**			+		
GST	35.7 ± 10.7	4.20 ± 5.35	80.6 ± 14.8	68.7 ± 15.5	2.94 ± 4.16	19.1 ± 9.4	108 ± 33	75.7 ± 9.5
		**	***	**	++	++		
APX	273 ± 54	464 ± 64	203 ± 14	313 ± 146	220 ± 56	552 ± 225	272 ± 27	307 ± 31
		**	*					

*, **, *** significant difference between the control and SA pre-, NaSA pre- or Cd-treated plants at the p < 0.05, 0.01 and 0.001 levels, respectively.

^**+**^, ^**++**^, ^**+++**^ significant difference between plants treated with Cd alone or in combination with SA or NaSA at the p < 0.05, 0.01 and 0.001 levels, respectively.

(SA pre: 0.5 mM SA pre-treatment for 1 day; NaSA pre: 0.5 mM NaSA pre-treatment for 1 day; Cd: 0.5 mM Cd for 1 day; SA pre Cd: 0.5 mM SA pre-treatment for 1 day followed by 0.5 mM Cd stress for 1 day; SA+Cd: addition of 0.5 mM SA and 0.5 mM Cd together for 1 day; NaSA pre Cd: 0.5 mM NaSA pre-treatment for 1 day followed by 0.5 mM Cd stress for 1 day; NaSA+Cd: addition of 0.5 mM NaSA and 0.5 mM Cd together for 1 day)

### GSH-Related Redox Changes

**Fig 7:**
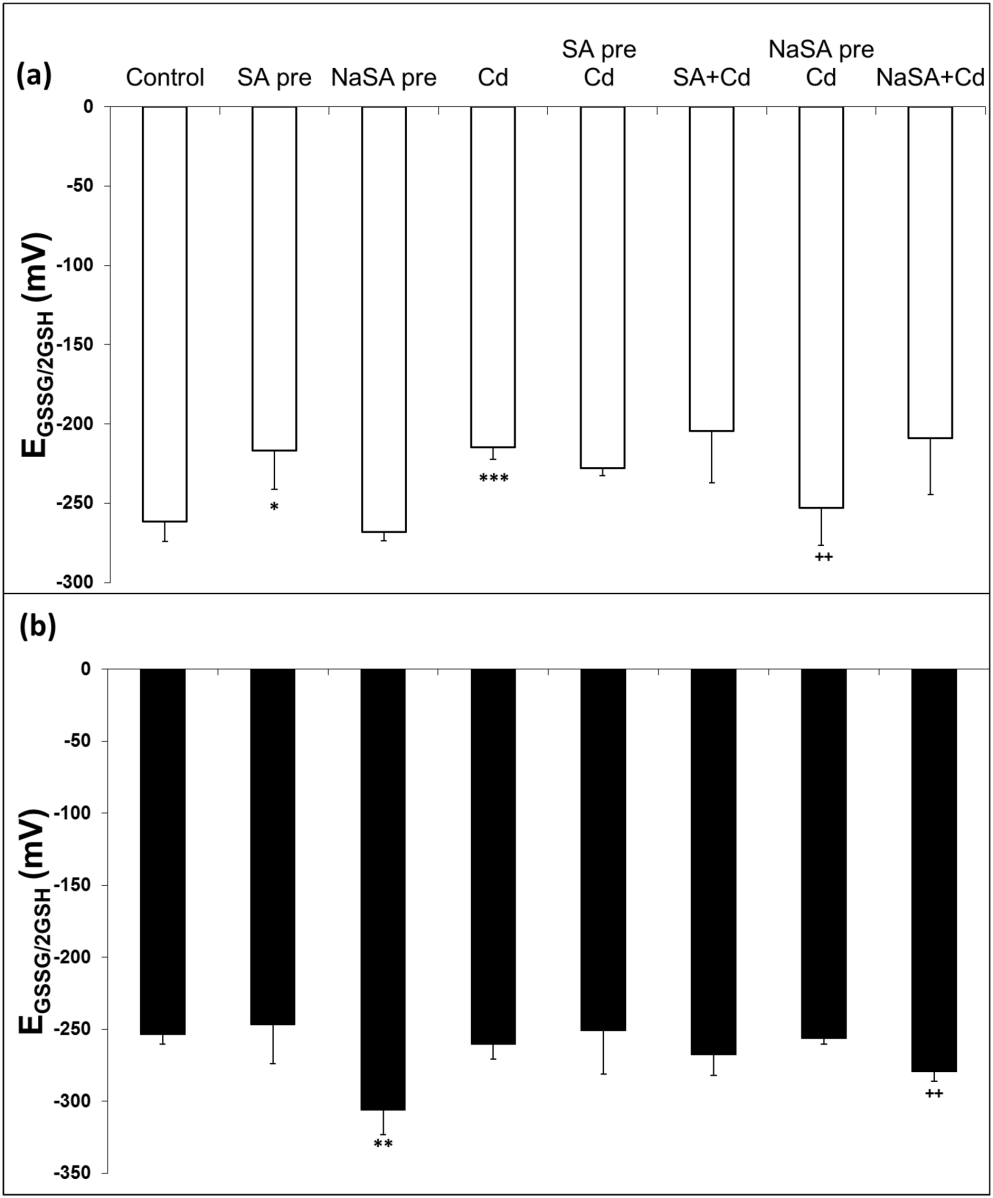
Changes in E_GSSG/2GSH_ in the roots (a) and leaves (b) of young maize plants after 0.5 mM SA or NaSA treatment during Cd stress. (For details see legend of Table 1).

**Fig 8:**
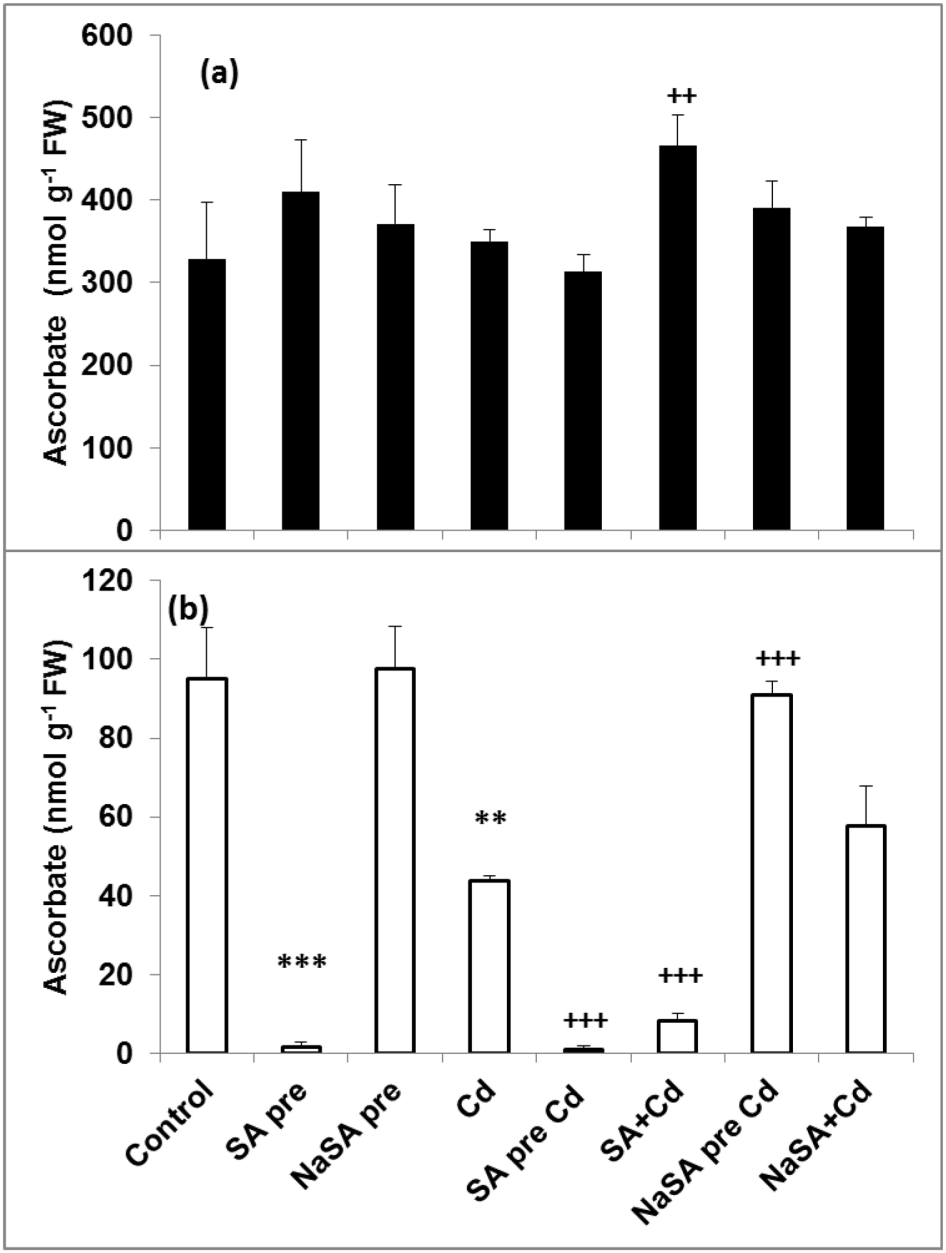
Changes in the AsA content in the leaves (a) and roots (b) of young maize plants after 0.5 mM SA or NaSA treatment during Cd stress. (For details see legend of Table 1).

The redox state of the plants was determined by calculating the E_GSSG/2GSH_ value of the roots and leaves. E_GSSG/2GSH_ increased in the roots after SA and Cd treatment compared with the control (Fig 7a), while no changes were detected in plants pre-treated with NaSA even after Cd addition. In contrast E_GSSG/2GSH_ did not change in the leaves of SA- and Cd-treated plants (Fig 7b), while it decreased after NaSA pre-treatment and when NaSA and Cd were applied together in comparison with the control. Glutathione reductase (GR) produces reduced GSH from the oxidised form and this enzyme is part of the ascorbate-glutathione cycle. The obtained results clearly indicated that SA and NaSA pre-treatment intensified the GR activity in the roots compared with the control (Table 2). After Cd treatment a rise was only observed when SA was applied at the same time as Cd in comparison with the Cd treated roots. The GR activity increased after pre-treatment with SA in the leaves compared with the control plants and it was enhanced after Cd addition. An increase in the GR activity was observed in the leaves of Cd, SA+Cd and NaSA pre Cd treated plants. Glutathione-S-transferase (GST) uses GSH for the detoxification of harmful compounds. GST activity diminished in the roots of plants pre-treated with SA and became even lower when the plants were treated with Cd (Table 2). NaSA pre-treatment caused a significant increase in GST activity compared with the control, and this level remained high after Cd treatment. No changes were detected in the leaves after SA or NaSA pre-treatment, but the GST activity dropped after Cd addition compared with the control except in the case of SA pre-treatment. The activity of ascorbate peroxidase (APX), another enzyme involved in the ascorbate-glutathione cycle, increased after SA pre-treatment but decreased in the roots of plants pre-treated with NaSA compared with the control (Table 2). Cd treatment did not affect the APX activity in the roots in the majority of cases (Table 2), but the activity was doubled when SA and Cd were added together. The APX activity rose in the leaves after Cd treatment, but remained at the control level in plants pre-treated with NaSA. The total AsA content did not change substantially in the leaves (Fig 8a). However, its content in the roots declined after SA pre-treatment compared with the control (Fig 8b). Cd treatment itself reduced the AsA content to half of the one of the control roots. Similar changes in the level of AsA were observed in the roots after Cd treatment when NaSA and Cd were added together (Fig 8b). The AsA content in the roots of plants pre-treated with NaSA remained at the control level even after Cd treatment.

## Discussion

SA has been shown to alleviate the harmful effects of Cd in maize and pea plants [12, 13, 29]. However, SA can also be a stress factor inducing oxidative stress when its concentration differs from the optimum level. In the present work it was demonstrated that the effect of exogenous SA depends greatly on its form, i.e. whether it is applied as an acid (SA) or a Na salt (NaSA). The mode of application may also be important, since different combinations may result in fundamentally different effects even at the same concentration. Fluorescence measurements showed that pre-treatment with NaSA in hydroponic solution protected maize plants more efficiently than SA (Table 1), which proved to be more stressful than NaSA or even than Cd treatment. When NaSA was applied simultaneously with Cd, it was less effective than in the case of pre-treatment, while plants treated with SA + Cd together performed better than plants treated only with Cd.

The role of SA in reducing the uptake and accumulation of Cd in plants has also been reported. The addition of sulpho-SA to rice seedlings decreased the accumulation of Cd in both roots and shoots [30]. In the present experiments both SA and NaSA decreased the Cd uptake of maize roots. Interestingly, when SA and Cd were added together, less Cd was accumulated in the root but several times more Cd was transferred to the leaves than when no SA treatment was applied. The differences were far less pronounced in the case of NaSA. The Cd uptake depends on the pH of the culture medium. It was found earlier that the amount of Cd accumulated by various plants was greater at higher pH values [31]. In the present experiment when Cd was added simultaneously with SA the pH of the growth solution was lower than in the other treatments (see S1 Fig), which may be the reason for the lower Cd uptake. However, neither the addition of NaSA to the solution nor pre-treatment with SA or NaSA significantly changed the pH of the growth medium, so the obtained results could not be explained by the effect of pH.

PCs have an important role in the detoxification of harmful ions by transferring metal–PC complexes into the vacuoles, thus sequestering the metals from sensitive enzymes. Although higher PCS activity was detected after Cd treatment in the roots of plants pre-treated with SA, the highest PC accumulation in the roots was found after treatment with NaSA. The highest level of PCs in the leaves was also detected in these plants, suggesting that the PCs synthesized in the roots are transferred to the leaves, which also exhibited elevated PCS activity and the highest Cd concentration. A large quantity of PCs was also found in the leaves when Cd and SA were added together, but this went hand in hand with increased PCS activity, so these PCs were most probably synthesized in the leaves.

In addition of being the precursor for PCs synthesis, the GHS plays an important role in the phytoextraction of toxic metals in plants via its involvement in metal chelation, compartmentation, homeostasis, antioxidative defence and signal transduction [32]. In fact, GHS effectively chelates Cd in living organisms to give the non-toxic form Cd(GS)_2_ [33]. Generally, the γECS enzyme is more important for the control of GSH synthesis than GSHS [34]. However, in *Lycium chinense* plants subjected to Cd stress conditions the γECS gene was not affected by endogenous SA, while the *LcGSHS* transcript levels were significantly increased together with a rise in the GSHS activity [35]. This increase in *LcGSHS* transcript levels was repressed when a 2-aminoindan-2-phosphonic acid, AIP (an inhibitor of SA biosynthesis) was added to the plants. This suggests that the expression of the Cd-induced *LcGSHS* transcript in *L*. *chinense* is controlled, at least in part, by an endogenous SA-dependent pathway [35]. Other studies also found that in the presence of Cd, GSHS is the rate-limiting enzyme for the synthesis of GSH in *B*. *juncea* [36]. In the present case both the γECS and GSHS activities increased after Cd treatment, but only in the roots of NaSA-treated plants. The GSH level in the roots rose in response to NaSA pre-treatment without Cd, but dropped after Cd treatment. This GSH was probably used for PC synthesis. The root γEC level increased in Cd-treated plants and in those treated with NaSA. It can be concluded that GSH synthesis in young maize plants under Cd stress was influenced by NaSA but not by SA.

Earlier results also suggested that SA treatment led to a decrease in oxidative injuries induced by Cd [12, 13, 37]. The production of H_2_O_2_ during abiotic stress serves as part of the signalling cascade, and increased levels of ROS might interact with SA. In the present experiment, although Cd substantially affected the photosynthetic processes, the MDA content in the leaves was not increased significantly. However, significant changes were observed in the antioxidant systems, indicating the induction of redox signalling. SA treatment decreased the MDA content in the roots even after Cd treatment, while NaSA increased it. SA pre-treatment caused a dramatic increase in the CAT activity, while NaSA triggered moderate activity, which was further increased after Cd treatment. In agreement with recent results, where SA promoted POD activity, possibly enhancing the tolerance of rice plants against Cd [30], POD activity also increased in SA-treated maize plants, while it decreased in NaSA treated plants. These changes could explain the different levels of oxidative stress in the roots of plants treated with different forms of SA.

Nevertheless, not only CAT and POD are responsible for the redox balance in plants. A high level of endogenous ascorbate is essential to maintain the antioxidant capacity if plants are to be protected from oxidative damage. In the present case both SA treatment and Cd stress diminished the level of AsA, while it remained unaltered in plants pre-treated with NaSA. A certain level of GSH is necessary in plant cells for the reduction of AsA and this is generated from GSSG by GR. A lower GSH/GSSG ratio was found in wild-type *Arabidopsis* plants than in SA-deficient transgenic lines [38]. In contrast earlier results showed that a higher endogenous SA level in the roots of Cd-tolerant wheat varieties increased the GSH/GSSG ratio during Cd stress, probably *via* an increase in GR activity, thus stimulating the antioxidant and metal detoxification systems and increasing Cd stress tolerance in wheat seedlings [39] In the present work the highest GR activity was recorded in the roots of plants pre-treated with NaSA, resulting in an extremely high GSH/GSSG ratio. The activity of GST, another GSH-related enzyme, also increased after NaSA or Cd treatment, while it decreased when SA was used. Changes in the content of glutathione or in the ratio between its reduced and oxidised forms affect the cellular reducing capacity and half-cell reduction potential, which can be used as stress markers [22]. There was no change in E_GSSG/2GSH_ in plants given NaSA pre-treatment compared with the control, while it increased after both Cd and SA treatment. The lower values of E_GSSG/2GSH_ recorded after NaSA pre-treatment could be an indication of Cd tolerance, since it has recently been shown that the E_GSSG/2GSH_ values of Cd-tolerant and accumulator *E*. *canadensis* plants were more negative than those of sensitive species even after Cd treatment [40]. These results suggest that GSH-related antioxidant enzymes are promoted by NaSA, while the other antioxidant enzymes may be influenced by SA.

## Conclusions

Under conditions of Cd stress, both SA and NaSA induce various defence mechanisms; however, their effectiveness and mechanisms of action are different. In the present experimental set up, a NaSA treatment preceding the Cd exposure of maize plants provided the best protection toward the stress conditions induced by the presence of Cd. SA and NaSA may affect antioxidant systems in different ways. The influence of SA and NaSA on the distribution of the heavy metal Cd may also differ. SA mainly facilitated the transport of Cd to the leaves, while NaSA mainly increased the PC level in the roots.

The exact reasons for the different effects of SA and NaSA are still unknown. As one is an acid and the other a salt, the pH or ionic strength of the medium could be involved. However, neither adding NaSA to the solution nor pre-treatment with SA or NaSA significantly changed the pH of the growth medium in the present case, so the differences could not be explained by the effect of pH.

The present results pointed out the differences in the mode of action of the acidic and salt forms of SA in plant protection against Cd stress. These findings highlighted the necessity to explicitly indicate the SA form that has been supplied to the plants in order to correctly interpret the obtained results and gain further insights into the SA-related mechanisms of Cd tolerance in plants.

## Supporting Information

S1 Fig Schematic presentation of SA and NaSA treatments. (TIFF)
