# Supplementary figures and images for: Salicylic Acid and Sodium Salicylate Alleviate Cadmium Toxicity to Different Extents in Maize (Zea mays L.)
Source: PLoS One. 2016 Aug 4;11(8):e0160157. doi: 10.1371/journal.pone.0160157 (PMC4973972; doi:10.1371/journal.pone.0160157)

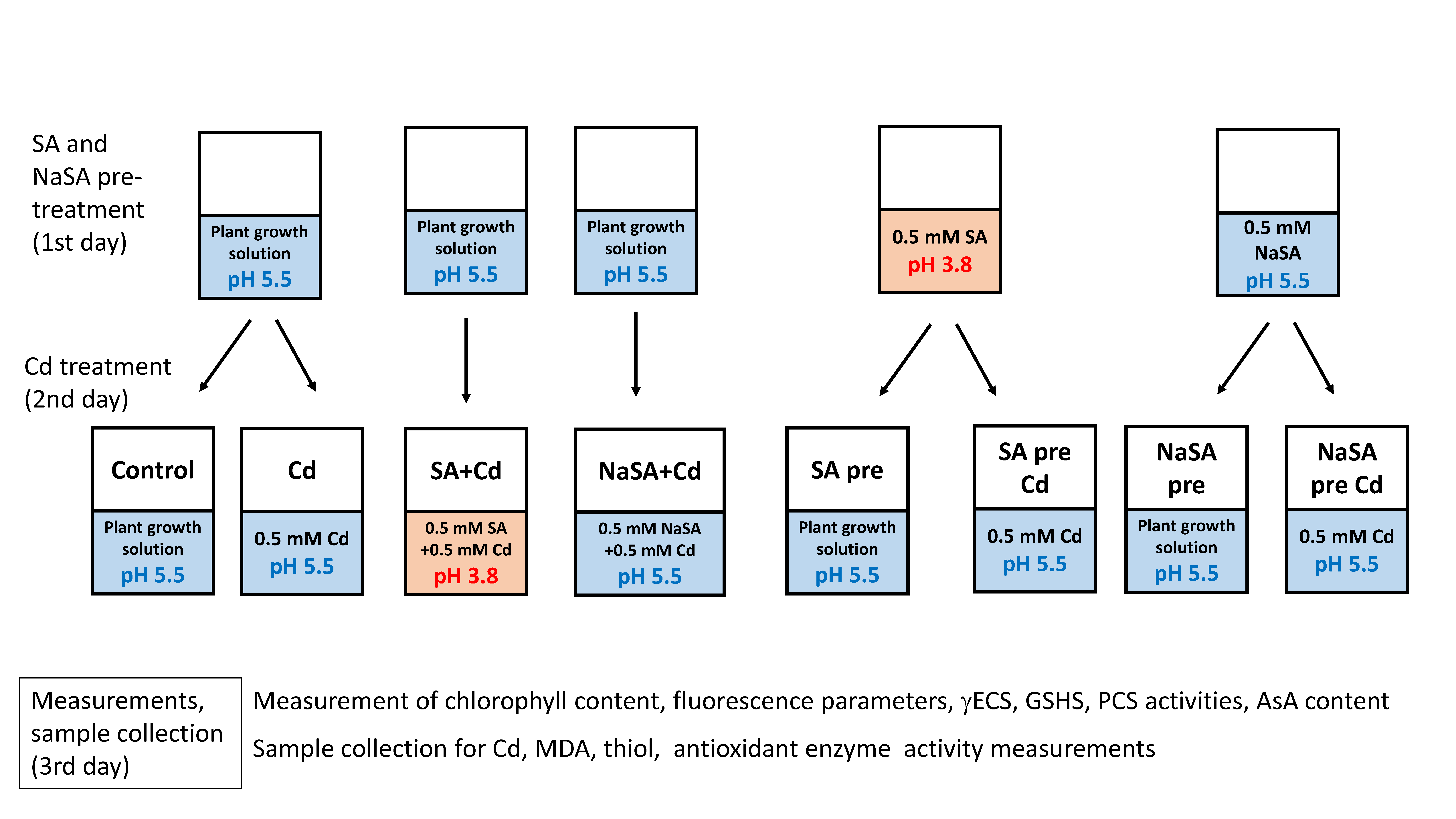

Supplement: S1 Fig — (TIFF) [file pone.0160157.s001.tiff]
